# Dicationic Ionic Liquids as Antibacterial and Conductive Plasticizers: Effect of Cationic Structures on Starch Film Properties for Flexible Electronics

**DOI:** 10.1021/acsabm.5c01229

**Published:** 2025-08-30

**Authors:** Susanna Romano, Benedetta Brugnoli, Serena De Santis, Daniele Rocco, Chiara Frezza, Giovanni Sotgiu, Giorgia Fiori, Gabriele Bocchetta, Salvatore Andrea Sciuto, Andrea Scorza, Irene Bavasso, Alessandro Stuart Savoia, Monica Orsini

**Affiliations:** † Department of Industrial, Electronic and Mechanical Engineering, 19012 Roma Tre University, Via Vito Volterra 62, 00146 Rome, Italy; ‡ Department of Chemistry, 9311 University of Rome “La Sapienza”, Piazzale Aldo Moro 5, 00185 Rome, Italy; § Department of Chemical Engineering Materials Environment, University of Rome “La Sapienza”, 00184 Roma, Italy

**Keywords:** starch films, dicationic ionic liquids, cationic effect, conductive films, flexible electronics

## Abstract

In the flexible electronics field, the growing issue of electronic waste and the massive use of nonbiodegradable substrates have led research toward sustainable materials based on natural polymers such as starch. Nevertheless, it lacks the specific properties of a processable plastic material and has no appreciable conductivity. Therefore, the use of appropriate plasticizers is necessary. Dicationic ionic liquids (DILs), characterized by good conductivity and lower toxicity compared with monocationic ILs, may represent a valid suggestion. In addition, DILs show greater antibacterial efficacy, which is particularly suitable for the production of wearable devices. This work investigates the role of the cationic structure of DILs in the characteristics of flexible starch films with both conductive and antibacterial properties. Four 1-ethyl-3-methyl imidazolium-based DILs with varying chain linkers were used to prepare starch films via solution casting. The study examined the impact of these plasticizers on the films’ mechanical properties, thermal stability, wettability, electrical conductivity, and antimicrobial activity. The prepared films were tested as materials for making wearable strain sensors, suggesting potential applications in the field of flexible electronics.

## Graphical abstract

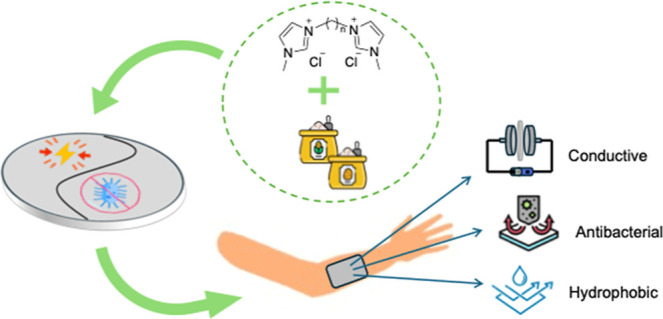

## 1 Introduction

In electronics, there is a growing demand for devices that can perform under dynamic and often complex conditions. These specific needs have led to the development of flexible electronics that offer features that rigid standard electronics do not allow. In the development of flexible and portable devices, the choice of the material matrix is crucial. The materials that can be building blocks of flexible electronic devices need to bend, roll up, fold, or stretch, in addition to showing suitable conductivity. Various properly prepared polymeric materials, including several hydrogels, have been tested.^1^ However, a key aspect to consider in the design and production of these materials, besides flexibility and conductivity, is their possible biodegradability. Flexible wearable technology, indeed, has become an integral part of our daily lives, providing unprecedented access to real-time health data, personalized fitness tracking, and seamless connectivity.^2^ As a result, this rapid integration of wearables into our routines has raised concerns about the environmental impact of the materials used in their manufacture.^3^ The proliferation of electronic waste from obsolete and discarded devices has spurred a revaluation of the sustainability of wearable technology.^4^ Biopolymer materials emerge as a promising alternative with attractive benefits to address this challenge.^5^ Among them, starch is a naturally abundant polysaccharide that stands out as one of the most potent naturally degradable materials globally.^6^ With wide-ranging sources, cost-effectiveness, and favorable thermodynamic properties, starch-based biodegradable materials find applications across various fields, including food packaging, agricultural production, papermaking, electronic devices, and more.^7^ However, native starch does not have the specific properties of a processable plastic material and has no appreciable conductivity. Among several possibilities to conveniently modify it, a straightforward procedure involves using an appropriate plasticizer. In recent years, ionic liquids (ILs) have been fundamental in the plasticization of starch, weakening the interactions among the polymer chains in order to yield thermoplastic starch.^8^ ILs are molten salts maintained in a liquid state below 100 °C, involving an organic cation paired with an organic or inorganic anion.^9^ Due to their intrinsic nature, ILs confer ionic conductivity to the produced starch-based materials.^10^ By variation of the anion–cation combinations, the properties of ILs can be tuned. As a result, ILs used as plasticizers have been tailored, allowing precise control over the conductive and mechanical properties of the obtained bioplastics.^11^ In recent times, dicationic ILs (DILs), a new category of the IL family, have attracted great attention. These ionic salts consist of two distal cationic head groups connected by a linking fragment, which can be a simple alkyl chain or a more functional structure and associated with two counteranions. Thanks to the possibility of selecting the type of the two head cationic groups that can be identical or different, choosing the nature of the binding fragments, and varying the anions, a large number of DILs have been synthesized.^12^ This wide structural variability, therefore, allowed for more adjustable and broader physical and chemical properties in comparison to those of monocationic ILs. In particular, compared to analogous monocationic ILs, symmetrical dicationic imidazolium-based ILs have demonstrated markedly lower toxicity^13^ and increased antibacterial efficacy.^14,15^ Specifically, incorporating longer alkyl chains onto the imidazolium cation enhanced activity, thereby reducing the minimal inhibitory concentration (MIC) against microorganisms.^16,17^

Previously, we have shown that symmetrical dicationic imidazolium-based ILs can be used as plasticizers to obtain conductive starch-based films.^18^ Hence, the idea of exploiting both their excellent antibacterial activity and their plasticizing ability. Biodegradable films that exhibit combined antibacterial and conductive properties are particularly suitable for the production of wearable devices. In fact, given the prolonged and intimate contact between wearables and the human body, incorporating antimicrobial properties becomes fundamental for user well-being.^19^ In this context, we aimed at fabricating starch-based antibacterial and conductive films for potential use in strain sensor.^20^ To the best of our knowledge, this is the first time that the effect of the different structures of dicationic imidazolium-based ILs on the properties of the resulting flexible starch films has been evaluated. Analysis of the correlation between DILs’ structure and the properties of the starch films produced can help design starch-based materials tailored to the application.

With this purpose, we prepared flexible thermoplastic starch films (TPS) by a solution casting method using four symmetrical 1-ethyl-3-methyl imidazolium-based dicationic liquids as plasticizers. The influence of the chain linkers’ length and the compositions (−CH_2_–, –(CH_2_)_5_–, –CH_2_C_6_H_4_CH_2_–, and –CH_2_(CH_2_OCH_2_)_3_CH_2_−) on the mechanical properties, electrical conductivity, antimicrobial activity, thermal stability, and hydrophobicity of the films was examined. In addition, the behavior of prepared films as suitable materials to produce strain sensors was considered.

## 2 Experimental Section

### 2.1 Materials

Arrowroot starch (16–27% content of amylose), 1-methylimidazole (Alfa Aesar), dimethyl sulfoxide (DMSO, Sigma-Aldrich), diethyl ether (Sigma-Aldrich), toluene (Sigma-Aldrich), dichloromethane (CH_2_Cl_2_, Sigma-Aldrich), 1,5-dichloropentane ((CH_2_)_5_Cl_2_, Sigma-Aldrich), α,α′-dichloro-*p*-xylene (Sigma-Aldrich), and bis­[2-(2-chloroethoxy)­ethyl] ether (Sigma-Aldrich) were used as received.

### 2.2 Synthesis of Imidazolium-Based DILs

All ^1^H NMR and ^13^C NMR spectra were recorded in DMSO solvent at 400 MHz and given in the Supporting Information (Figures S1–S6). ^1^H NMR and ^13^C NMR peaks confirm the synthesis of imidazolium-based DILs.

#### 2.2.1 Synthesis of 3,3′-Methylenebis­(1-methyl-1*H*-imidazole-3-ium) Dichloride

The preparation of the DIL (C_1_H_2_(MIm)_2_ Cl_2_) was performed following the same procedure described in our previous work.^18^ Briefly, in a round-bottom flask, 1-methylimidazole (20 mmol) and CH_2_Cl_2_ (60 mmol) were added together in 1.15 mL of DMSO. The reaction was conducted under stirring at 95 °C for 24 h. The resulting white insoluble precipitate was subjected to three washes with diethyl acetate to eliminate DMSO and any residual unreacted imidazole. The product was dried in a rotavapor without requiring additional purification.

#### 2.2.2 Synthesis of 1,5-Bis­(1-methyl-1*H*-imidazole-3-ium) Pentane Dichloride

The synthesis of C_5_H_10_(MIm)_2_Cl_2_ was conducted under the same experimental conditions and procedure used for C_1_H_2_(MIm)_2_ Cl_2_, where 50 mmol of 1-methylimidazole and 25 mmol of (CH_2_)_5_Cl_2_ were added in 1.2 mL of DMSO. The product was dried in a rotavapor without the need of additional purification steps.

#### 2.2.3 Synthesis of 3,3′-Bis­(1-methyl-1*H*-imidazole-3-ium) 1,3-Phenylenedimethylene Dichloride

The preparation of C_6_H_4_(CH_2_MIm)_2_Cl_2_ was performed with slight changes from the method described by Dutta et al.^21^ In a round-bottom flask, 20 mmol of 1-methylimidazole was added to 10 mmol of α,α′-dichloro-*p*-xylene in 4 mL of toluene. The reaction was conducted at 100 °C for 24 h. The recovery of the product followed the same procedure used for the previous dicationic salts.

#### 2.2.4 Synthesis of 1,11-Bis­(1-methyl-1*H*-imidazole-3-ium) (3,6,9-Trioxaundecane) Dichloride

The synthesis of C_8_H_16_O_3_(MIm)_2_Cl_2_ was performed with slight changes from the method described by Zare et al.^22^ In a round-bottom flask, 1-methylimidazole (50 mmol) and bis­[2-(2-chloroethoxy) ethyl] ether (25 mmol) were mixed in 1.2 mL of DMSO. The reaction proceeds for 24 h at 95 °C. The yellowish product was dissolved in a limited quantity of methanol and then precipitated in ethyl acetate. Following the removal of ethyl acetate through decantation, the resulting yellowish product was dried in a rotavapor.

### 2.3 Bacterial Strains

*Staphyloccocus epidermidis* ATCC 35984 (Gram-positive) and *Pseudomonas aeruginosa* PAO-1 (Gram-negative) were selected as strain models due to their association with infections on biomedical devices. Each strain was plated on agar and incubated overnight at 37 °C. Once discrete colonies were obtained, three of the isolated colonies were inoculated in 5 mL of their culture medium, Luria–Bertani broth (LB) for *P. aeruginosa* and Tryptone soya broth (TSB) for *S. epidermidis*, and left under agitation for 3 h instead of 24 h until the exponential growth phase was reached. Subsequently, cultures were diluted to obtain a final optical density at 600 nm (OD_600_) of 0.05 for the MIC and bacterial growth curve inhibition, respectively.

#### 2.3.1 Microbial Inhibition Concentration of DILs

The microbial inhibition concentration (MIC) value of each DIL was determined by the broth microdilution method. First, each DIL was solubilized in sterile water (100 mg/mL) and then filtered at 0.2 μm (Artiglass). 2-fold serial dilutions of each DIL were made in Mueller–Hinton (MH) broth (100 μL) in a 96-well microtiter plate over the range of 200–2 mM. Overnight cultures of each bacterial strain (100 μL) were added to the wells and diluted to an OD_600_ = 0.05. As a negative control, 100 μL of sterile LB or TSB was added to 100 μL of MH broth. Instead, 100 μL of each inoculum was added to 100 μL of MH broth as a positive control. Each plate was incubated for 24 h at 37 °C. MIC values were chosen considering the absence of turbidity and measured using a microplate reader (λ = 620 nm). Results are reported in Table S1.

### 2.4 Preparation of Starch Films

Starch films (TPS) were produced by using the solvent casting and evaporation technique. Initially, 1 g of arrowroot starch was dissolved in 30 mL of distilled water and stirred for 30 min, achieving complete gelatinization at 95 °C. Subsequently, 0.4 g of each DIL plasticizer was added into the solution and heated for 10 min at the same temperature until a homogeneous mixture was obtained. The solution was then cooled to 65 °C, poured into a silicone Petri dish with a diameter of 90 mm, dried for 24 h in an oven at 45 °C, and finally left at room temperature for an additional 24 h. The dried films were peeled off from the casting plate and stored at 50% relative humidity (RH). The list of prepared films is shown in Table 1.

**Table 1:** Composition of the Prepared Films

acronym	plasticizer
TPS_D1	C_1_H_2_(MIm)_2_ Cl_2_
TPS_D2	C_5_H_10_(MIm)_2_ Cl_2_
TPS_D3	C_6_H_4_(CH_2_MIm)_2_Cl_2_
TPS_D4	C_8_H_16_O_3_(MIm)_2_Cl_2_

### 2.5 Film Characterizations

#### 2.5.1 Film Thickness

The film’s thickness was assessed using a digital micrometer, with three measurements taken at three separate points for each film.

#### 2.5.2 Moisture Content

The total amount of moisture absorbed into the films, conditioned at 50% of RH, was calculated gravimetrically through the following eq 1:

1 $$moisture\;content\;\left(MC\right)\;\%=\frac{\left(M_{i}-M_{f}\right)}{M_{i}}\times 100$$

where *M*_*i*_ and *M*_f_ are the weights of the films before and after drying at 105 °C for 24 h.

#### 2.5.3 Stability in Artificial Sweat

The film stability in artificial sweat, prepared according to the ISO standard ISO 3160-2, was assessed following Anyango et al.^23^ procedure with some modification. Films (2 cm × 2 cm) were dried in an oven at 100 °C for 2 h and then weighted. The dried films were immersed in 10 mL of artificial sweat solution and left for 7 days. After this time, the films were dried in an oven at the same conditions as before and weighed (*W*_f_). The weight loss is calculated through eq 2:

2 $$\%\;weight\;loss=\frac{W_{i}-W_{f}}{W_{i}}\times 100$$

#### 2.5.4 Scanning Electron Microscopy

A scanning electron microscope (SEM, MIRA3 by Tescan, Brno, Czech Republic), operated at 5.0 kV, was employed to examine the morphology of the films. For all the images, a magnification of 1000× was used. The film samples were mounted on aluminum stubs and fixed with double-sided adhesive tape. Subsequently, the samples were coated with a thin golden layer to prevent charging.

#### 2.5.5 Fourier-Transform Infrared Spectroscopy

FTIR spectra in the 4000–650 cm^–1^ region were acquired utilizing a Nicolet iN10 IR microscope (Thermo Fisher Scientific IT, Milano, Italy) featuring a mercury–cadmium–telluride (MCT-A) nitrogen-cooled detector in the ATR mode. Each spectrum was generated by averaging 64 interferograms and applying a Blackman-Harris correction for apodization. The background spectrum was obtained in ambient air. The nominal spectral resolution was set at 8 cm^–1^. Data acquisition and spectral processing were conducted using OMNIC SPECTA software provided by Thermo Fisher Scientific.

#### 2.5.6 Thermogravimetric Analysis

The thermal stability of starch films was assessed by using thermogravimetric analysis (TGA) with a Mettler TG 50 thermobalance. The analysis involved a thermal scanning range of 25–500 °C and a scanning speed of 10 °C/min. Under a nitrogen flow, samples weighing 5–6 mg were analyzed. The degradation temperature was identified at the peak of the first derivative of weight concerning the temperature.

#### 2.5.7 Differential Scanning Calorimetry

Thermal properties of the cast films (conditioned at an RH of 50%) were evaluated by differential scanning calorimetry (DSC) (Mettler Toledo DSC 822e). All of the analyses were carried out under a nitrogen flow (50 mL min^–1^) on about 5 mg of sample. The glass transition temperature was defined as the midpoint of the increase in heat capacity. The applied temperature program is as follows: first, the samples were placed on opened capsules, cooled to −70 °C, and then a temperature ramp up to 180 °C at 10 °C min^–1^ was applied, detecting water evaporation. Subsequently, the dry samples were first cooled to 20 °C and then heated from 20 up to 250 °C at 10 °C min^–1^. The glass transition temperatures were reported as dry *T*_g_.

#### 2.5.8 X-ray Diffraction Analysis

XRD analyses were performed on film samples (stored at room temperature at 50% RH) at ambient conditions by an XRD apparatus (Smartlab SE, Rigaku Corporation, Tokyo, Japan) with a Cu–Kα radiation source (λ = 1.54 Å) at 40 kV voltage and 50 mA current. Intensities were collected by step scanning in the 5°–40° (2θ) range with a step of 0.01° and a scan speed of 1.50°/min. The following eq 3 was used to estimate the crystallinity of the different samples:^24^

3 $$\chi_{c}=\frac{\sum_{i=1}^{n}A_{ci}}{A_{t}}$$

where *A*_*ci*_ is the area under each crystalline peak and *A*_t_ is the total area, both amorphous and crystalline, under the diffractogram.

#### 2.5.9 Static Contact Angle Determination and Topography Measurements

Contact angle measurements were performed utilizing the sessile drop method with an Attension Theta Optical Tensiometer (Biolin Scientific, Sweden/Finland). Each measurement spanned 3 s, during which 125–140 images were analyzed. The device automatically established the baseline during the measurements. The contact angle (θ) was determined by fitting the drop’s shape to the Young–Laplace equation.

Surface topography was characterized by digital holographic microscopy using a DHM-R2100 system (Lyncée Tec SA, Lausanne, Switzerland) operated in the reflection mode. Before the measurements, all films were conditioned for 24 h at 23 °C and 50% RH to match the conditions used for the contact angle experiments. Each TPS_DIL film was imaged in three randomly selected regions. Optical phase maps were acquired in the single wavelength configuration (λ = 666 nm) with automatic phase unwrapping and a 5× objective, resulting in a 0.5× 0.5 mm field of view. Raw phase data were converted to height information and analyzed with the Koala surface roughness module (Lyncée Tec) in order to compute the mean height Sa and root-mean-square height Sq roughness parameters.

#### 2.5.10 Antimicrobial Activity

Starch films were cut into disks (Ø = 6 mm) and immersed in plastic tubes containing 100 μL of inoculum and 4900 μL of broth (LB or TSB) to a final OD_600_ = 0.05. Positive controls were arranged by putting 100 μL of each inoculum to 4900 μL. Blanks were also made by adding 100 μL of each sample to 4900 μL of TSB or LB broth.

All the samples were incubated under agitation at 37 °C. Bacterial growth inhibition (BGI %) was calculated by measuring OD_600_ at 24 h using a spectrometer and calculated through eq 4:

$$BGI\;\left(\%\right)=1-\left(\frac{{OD}_{sample}-{OD}_{0}}{{OD}_{ctrl}-{OD}_{0}}\right)\times 100$$ 4

where OD_sample_ is the absorbance measured at the predetermined times.

#### 2.5.11 Mechanical Tests

The films’ mechanical properties, including tensile strength (TS), Young’s modulus (*E*), and elongation at break (ε%), were determined through tensile testing using an Instron 4502 instrument from Instron Inc. (Norwood, MA, USA). For the analysis, films stored at room temperature with 55% RH were cut into rectangular specimens measuring 63.5 mm × 9.53 mm × (0.120–0.200) mm. These specimens were securely clamped between two flat jaw grips, and measurements were conducted at a deformation rate of 10 mm min^–1^ with a 2 kN load cell.

#### 2.5.12 Electrochemical Properties

Ionic conductivity measurements were conducted using electrochemical impedance spectroscopy (EIS) with a polymer film situated between two blocking stainless steel metal electrodes featuring a combined surface area of 0.95 cm^2^. The Metrohm Autolab PGSTAT204 apparatus was employed, applying an AC amplitude of ±10 mV across a frequency range of 1 MHz–1 Hz. The starch film conductivity was assessed at room temperature under controlled humidity conditions and during a temperature scan (RH = 55%). The conductivity was calculated using eq 5 below, where *l* represents the film thickness, *A* denotes the film area, and *R* (Ω) represents the film resistance derived from the intercept of the Nyquist plot (*Z*′ vs *Z*″) with the real axis:

5 $$\sigma \left(S/cm\right)=\frac{l}{A\times R}$$

The working electrochemical window of the samples was measured using the linear sweep voltammetry (LSV) technique. The voltage applied increases from 0 to 3 V with the scan rate of 1 mV s^–1^.

The change in electrical resistance as a function of stretch was also evaluated. Samples of 5 × 0.5 cm were cut from each film and fixed to the forefinger of an operator by copper tape and insulating tape (Figure S7a). The two pieces of copper tape were used as electrical terminals to be connected to a digital multimeter (Yokogawa DM7560) for electrical resistance measurement. The same operator repeatedly performed a bending and relaxing movement of the finger under the same environmental and measurement conditions. The relaxation position, achieved with the finger outstretched, was taken as the initial position (Figure S7b), while the maximum bending position of the forefinger, assumed as the final position, was reached by grasping a 7 cm diameter cardboard cylinder (Figure S7c). Twenty finger-bending relaxation cycles were performed for each sample and acquired through the digital multimeter at a sampling rate of 20 Hz.

## 3 Results and Discussion

### 3.1 Preparation of Starch Films

To guarantee the production of stable and flexible starch film, 40 wt % of plasticizer with respect to the dry starch was selected.^25^ The films were prepared using a solution casting technique, and four DILs, C_1_H_2_(MIm)_2_Cl_2_, C_5_H_10_(MIm)_2_Cl_2_, C_6_H_4_(CH_2_MIm)_2_Cl_2_, and C_8_H_16_O_3_(MIm)_2_Cl_2_, were employed (Figure 1). Films plasticized with C_1_H_2_(MIm)_2_ Cl_2_ had already been prepared in our previous work.^18^

**Figure 1:**
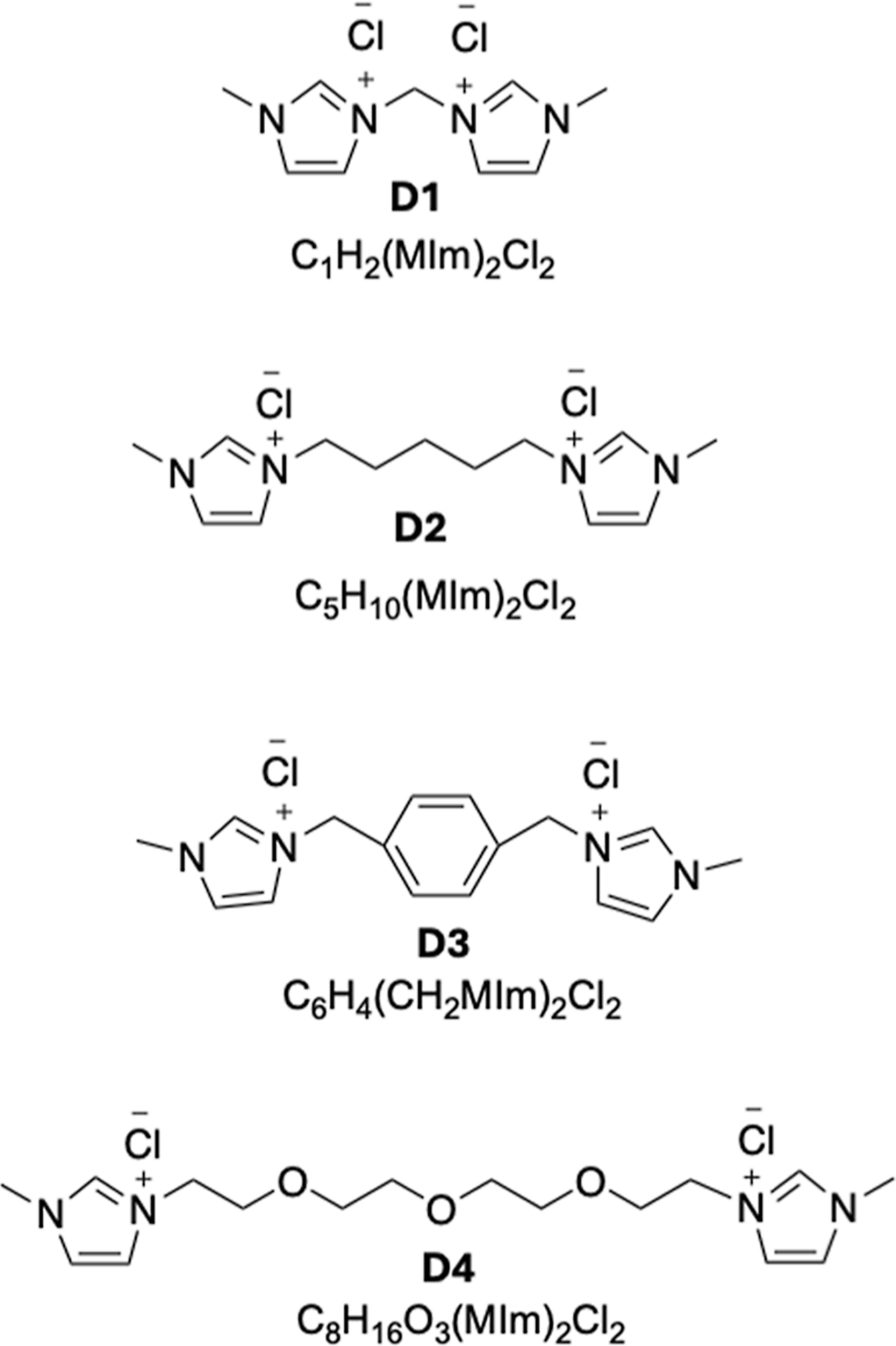
DILs used as plasticizers.

The properties of the obtained films were evaluated to find correlations between the structures of the cations’ linkers and their physical-chemical characteristics.

### 3.2 Appearance, Water Content, and Stability in Artificial Sweat

All prepared films presented similar thicknesses ranging from 0.17 to 0.20 mm, with a homogeneous surface without bubbles. They appeared white in color, displaying a good transparency, as the images reported in Figure S8 showed. The prepared films’ transparency confirmed the DILs’ ability to function as excellent starch plasticizers. The morphology of the film’s surface was evaluated by SEM (Figure 2). All the plasticized films showed a homogeneous surface, indicating good miscibility and the plasticizing effect of all the DILs.^26^ The micrographs did not reveal aggregation phenomena, defects, or pores, with slight surface heterogeneity when C_8_H_16_O_3_(MIm)_2_Cl_2_ is used (Figure 2D). The amount of water content resulted in comparable amounts within the different types of samples, obtaining for all the films a moisture content of MC % = 10% ± 1%.

**Figure 2:**
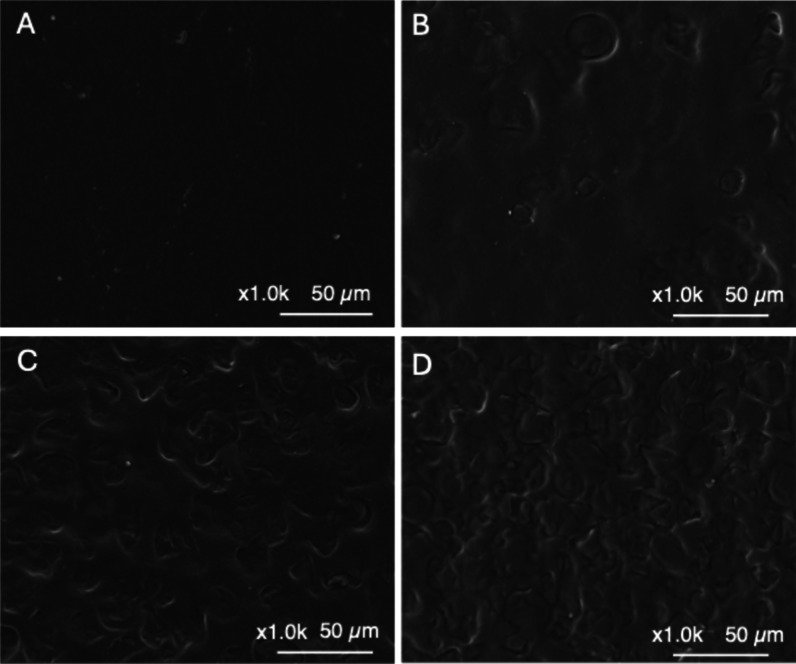
SEM images of surface morphology of (A) TPS_D1, (B) TPS_D2, (C) TPS_D3, and (D) TPS_D4.

The stability of the film in the artificial sweat solution was also evaluated. All samples remained intact with no visible changes. The percentage weight loss after 7 days immersion was determined to be approximately 20% ± 4% for all films (Table S1). Therefore, the moderate weight loss observed suggests that the films are sufficiently stable for wearable applications involving limited contact with the skin and limited exposure to sweat.

### 3.3 FTIR Analysis

FT-IR analyses were conducted to investigate the interactions between the plasticizers and starch. The spectra of the starch powder and the different DILs are reported in Supporting Information (Figure S9), and the spectra of the samples are presented in Figure 3. Notably, the film spectra displayed no indications of additional bands, signifying that the DILs did not undergo a reaction with the starch. All spectra exhibited absorption bands indicative of the starch structure, showing the C–O–C stretching in the 700–950 cm^–1^ region, signals associated with C–C, C–O stretching, and C–O–H bending in the 950–1200 cm^–1^ range, as well as a broad band between 3000 and 3600 cm^–1^, attributed to the stretching of O–H groups. The bands between 2800 cm^–1^ and 3000 cm^–1^ are characteristic of C–H stretching, and the signal at 1642 cm^–1^ is ascribed to the bending of absorbed water.^27^ Within the 3200–3400 cm^–1^ range, slight changes in the positions of vibrational bands associated with the stretching of OH groups were observed. These changes resulted from an interaction involving the starch, the introduced DIL, and the water molecules absorbed by the films. Specifically, when the O–H groups of starch engage in hydrogen bonding, the vibrational absorption band shifts toward lower wavenumbers. Conversely, a decrease in hydrogen bond strength leads to a shift in signals toward higher wavenumbers.^18^ Considering this phenomenon, the interactions between starch chains and various DILs primarily depend on the characteristics of the cationic moiety. With an increase in steric hindrance of cationic structure, a noticeable redshift in the absorption band is observed. This shift suggests a heightened capacity of the IL to weaken hydrogen bonding interactions among starch chains. Specifically, in the presence of plasticizer C_1_H_2_(MIm)_2_Cl_2_, the absorption band associated with OH stretching occurs at 3310 cm^–1^.^18^ As the alkyl chain length between the two imidazolium rings increases (C_5_H_10_(MIm)_2_Cl_2_), the stretching of OH shifted to 3314 cm^–1^, indicating greater spatial separation among the starch chains. Films plasticized with C_6_H_4_(CH_2_MIm)_2_Cl_2_ display an absorption band associated with the –OH stretching vibrations at 3321 cm^–1^. Therefore, when the distance between the two cation groups increases, a shift toward higher wavenumbers of OH vibrational mode is still observed even when the linking segment is nonlinear, as for the presence of the phenyl group. Subsequently, employing IL C_8_H_16_O_3_(MIm)_2_Cl_2_, characterized by a longer linear linker between imidazolium rings containing ether groups, results in a notable redshift of the –OH band, reaching 3332 cm^–1^. Based on the results from the IR analysis, the length of the segment linking the two cationic groups predominates in determining the interaction with starch chains, irrespective of the presence or absence of functional groups along this segment. This finding suggests that the spatial separation between the cationic groups exerts a more significant influence on their interaction with the starch network than do the specific chemical functionalities present within the connecting linker.

**Figure 3:**
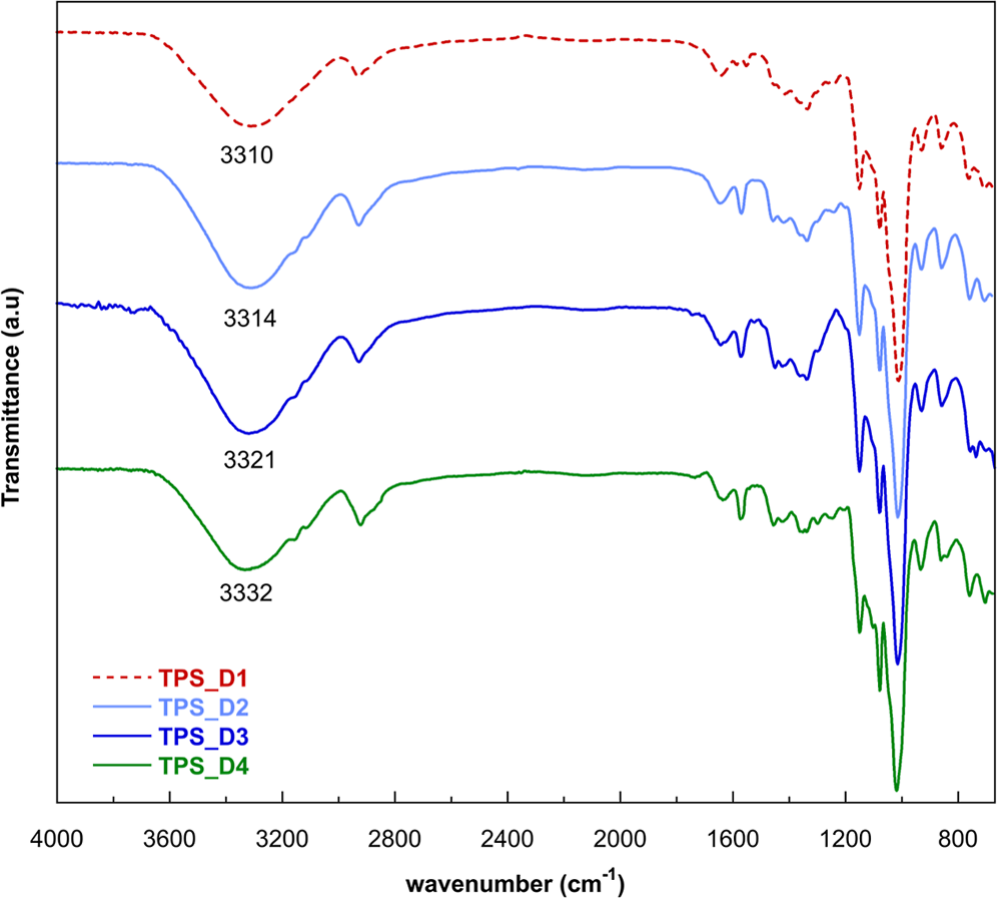
FTIR spectra of the different TPS_DIL films.

### 3.4 Thermogravimetric Analysis

TGA was employed to assess the thermal stability of the TPS films. Figure 4 illustrates the thermograms of the various starch films plasticized with DILs and a nonplasticized starch film, allowing for comparative analysis. Across all samples, an initial weight reduction occurred between 25 and 150 °C, which was attributed to the evaporation of absorbed moisture. The TPS film without plasticizers showed a decomposition temperature of 315 °C, while the plasticized films displayed a similar thermal decomposition profile, with degradation temperatures ranging from 245 to 260 °C. This result confirms our previous exploration,^18^ indicating that the thermal stability of starch films, when plasticized with ILs, primarily depends on the nature of the anion, while the influence from the cation remains minimal.

**Figure 4:**
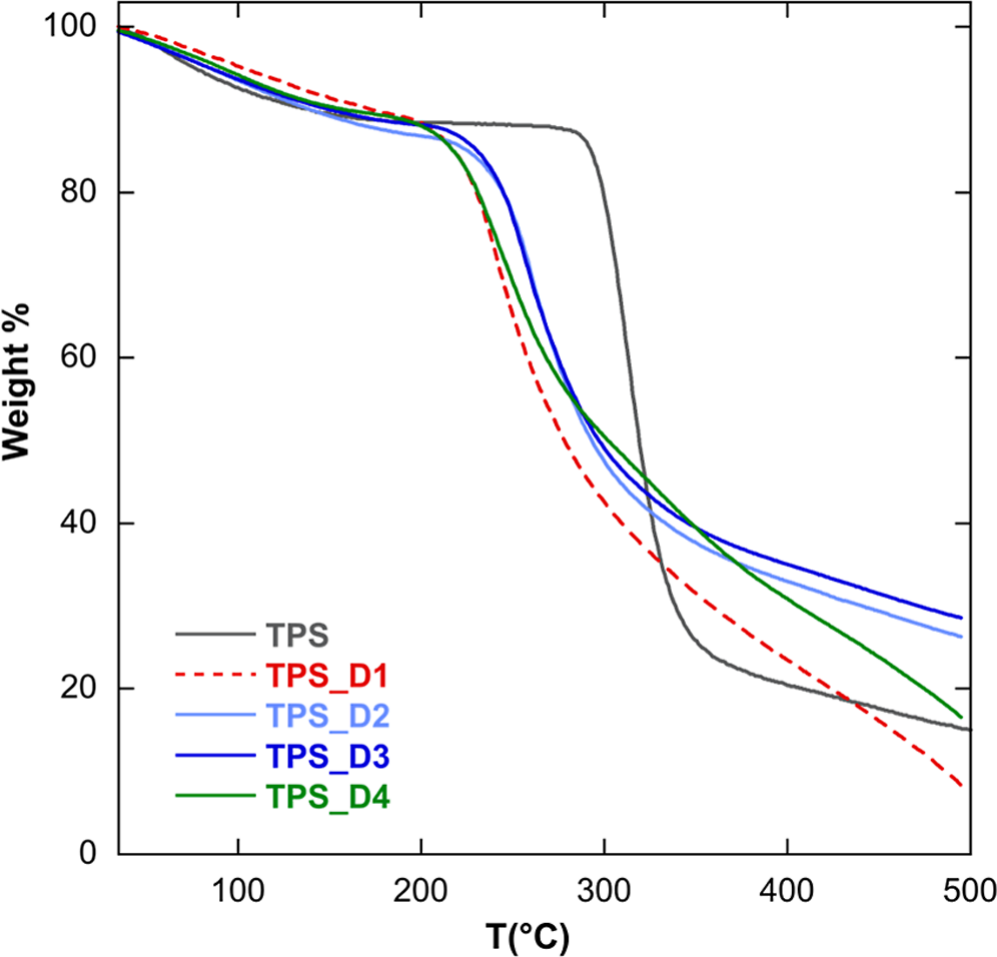
Thermogravimetric curves of TPS and TPS_DIL films.

### 3.5 Differential Scanning Calorimetry

Through DSC analysis, it was possible to determine the glass transition temperatures of the different films. The analyses were carried out on dried samples to minimize the effect of water and to assess the influence of ILs as plasticizers. The values of the transition temperatures (reported as dry *T*_g_) are shown in Table 2. All DILs showed good plasticizing abilities, as the *T*_g_ values of the TPS_DIL films were lower than the nonplasticized starch film (TPS). This is because plasticization, by reducing the intermolecular forces between the polymer chains and increasing their mobility, leads to a reduction in *T*_g_.^28^ Indeed, the glass transition temperature gradually decreases from around 121 °C (TPS) to the lowest temperature of 86 °C (TPS_D4). The evidence of this effect on the weakening of polymer chain interactions is in line with what has already been found from FTIR analysis, confirming that the length of the segment linking the two cationic groups greatly influences matrix interactions.

**Table 2:** Glass Transition Temperatures of the Different Dry TPS_DIL Films

sample	dry *T*_g_ (°C)
TPS	121 ± 4
TPS_D1	110 ± 2
TPS_D2	111 ± 1
TPS_D3	105 ± 1
TPS_D4	86 ± 3

### 3.6 X-ray Diffraction Analysis

XRD technique was employed to evaluate the crystallinity of the starch film samples. In native starch, three polymorphic structures, A-type, B-type, and V_H_-type, can be distinguished, each displaying typical XRD patterns.^29^ These distinct crystal structures can interact with water molecules in different ways. Both A-type and B-type structures are composed of double left-handed helices packed parallel in the crystal lattice. The A-type polymorph has a monoclinic unitary structure containing two double helices and four water molecules, while the B-type presents a hexagonal unit structure containing two double helices and 36 water molecules per unit cell. The V_H_-type polymorph, associated with amylose, is characterized by a single left-handed helix with a hydrophilic outer surface and a hydrophobic inner channel that can accept host molecules.^30^

The XRD patterns for the native starch and various plasticized starch films are shown in Figure 5. In the spectrum of the native starch, the main diffraction patterns are related to the typical A-type structure, with its characteristic diffraction peaks at 15° and 23° and the doublet at 17° and 18°. In the plasticized films, this typical doublet disappeared, revealing a loss of the A-type pattern. Instead, the plasticized samples showed the B-type pattern as indicated by the peaks at 17°, 22°, and 24° and a weak V_H_-type structure suggested by the presence of the peak at 20°. Figure 5 shows a table with the total crystallinity values and the respective fraction for type A, B, and V_H_ structures. For all films, the overall crystallinity was lower than that of native starch, showing that DILs act as good plasticizers. In detail, the films obtained with C_1_H_2_(MIm)_2_ Cl_2_ had a higher crystallinity content, while the other samples showed comparable crystallinity. These results are in agreement with observations by FTIR analysis, which revealed a rising distance between starch chains as the steric hindrance of the plasticizer molecules increases. The more disordered distribution can impede compact packing, reducing the crystallinity.

**Figure 5:**
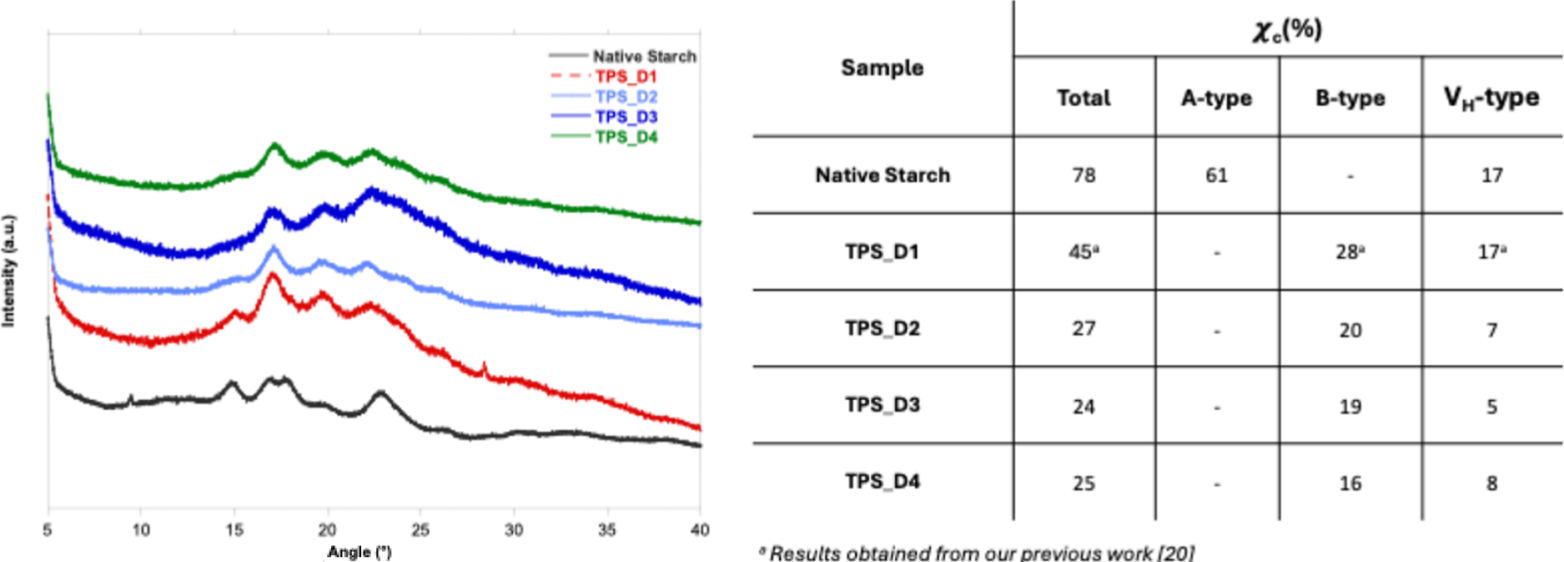
XRD patterns and parameters for the crystalline fraction of native starch and TPS_DIL films.

### 3.7 Mechanical Properties

Understanding the mechanical properties of starch films is pivotal in assessing their potential for prospective applications. These properties can be influenced by variables such as film thickness, the polymer network, and the type of plasticizer used.^31^ The mechanical property values, including the TS, elongation at break (ε%), and Young’s modulus (*E*), for the analyzed films are reported in Table 3, with stress–strain curves detailed in Supporting Information (Figure S10).

**Table 3:** Young’s Modulus (*E*), TS, and Elongation at Break (ε %) of TPS_DIL Films

sample	Young modulus (MPa)	Tensile strength (MPa)	ε (%)
TPS_D1	490 ± 60	11 ± 2	24 ± 7
TPS_D2	170 ± 20	6 ± 1	36 ± 5
TPS_D3	50 ± 8	4 ± 1	43 ± 3
TPS_D4	42 ± 3	3 ± 1	29 ± 4

The structure of the cation influences the mechanical properties of starch films. DILs inducing the more considerable weakening of interchain interactions, according to FTIR, are responsible for low values of Young’s modulus and TS values, indicating a direct correlation between the cationic structure and the mechanical behavior of the films.

When plasticizer C_1_H_2_(MIm)_2_ Cl_2_ is employed, the highest values of E and TS are observed, reaching 490 and 11 MPa, respectively. Increasing the carbon chain length of the linker (C_5_H_10_(MIm)_2_Cl_2_) results in decreased values, dropping to 170 and 6 MPa, respectively. The lowest values are possessed by films plasticized with C_6_H_4_(CH_2_MIm)_2_Cl_2_ and C_8_H_16_O_3_(MIm)_2_Cl_2_, with comparable results between them.

Similarly, elongation at break is affected by the characteristics of the plasticizing DIL, rising significantly as the length of the chain connecting the two cationic groups increases. Indeed, going from C_1_H_2_(MIm)_2_Cl_2_ to C_5_H_10_(MIm)_2_Cl_2_, ε% increases from 24% to 36%, comparable with the value of TPS_D4. The highest value of elongation at break is obtained for films plasticized with C_6_H_4_(CH_2_MIm)_2_Cl_2_. It can be deduced that the increase in steric hindrance of dication combined with the greater distance between the two imidazolium rings promotes chain mobility within the film matrix, resulting in increased flexibility. Therefore, understanding the relationships between cationic structure and mechanical properties is crucial for designing starch-based films with tailored functionalities for various electronic applications, such as motion sensors.^32^

### 3.8 Electrochemical Properties

The electrochemical properties of films are related to an interplay between polymer segment motions and ion–polymer interactions, which at the same time dissociate ions. As a result, various factors can affect the conductivity of these materials, including van der Waals forces, temperature, electrostatic forces, interaction between cations and anions, and the water content.^33^ The electrochemical stability window is an important parameter for a material that could be used in flexible electronic devices. To investigate the decomposition voltage of the studied films, LSV measurements were performed.^34,35^ Figure 6 presents the current vs voltage plot for all of the samples. TPS_D3 and TPS_D4 samples showed a similar behavior, with the current increasing significantly when the potential reaches 1.35 and 1.40 V, respectively, and indicating the electrolyte decomposition. TPS_D2 film showed a voltage breakdown slightly higher of 1.6 V, while TPS_D1 had the higher values of around 1.8 V. A working potential above 1 V allows the application of such materials in flexible supercapacitors based on biodegradable components.^36^

**Figure 6:**
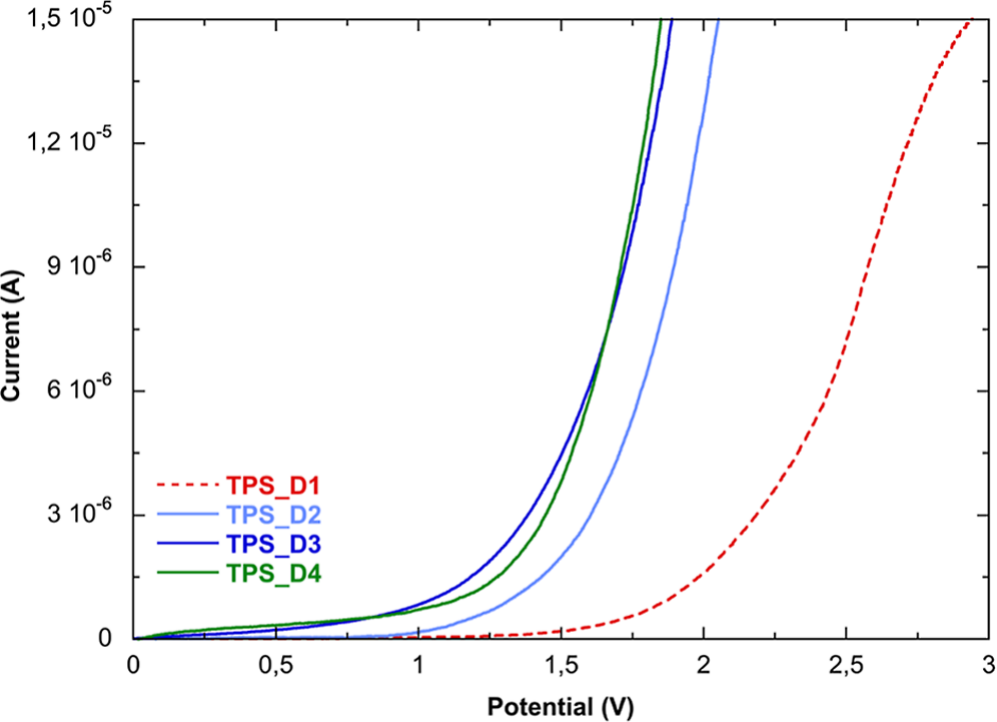
Electrochemical stability windows of TPS_DIL films.

The ionic conductivity of the different starch films was investigated through EIS. Since the conductivity of these membranes can be significantly affected by ambient moisture, systematic characterization at different humidity levels provides valuable insights into their performance. The room temperature ionic conductivity of the samples at different RH conditions (55%, 69%, and 84%) is reported in Table 4.

**Table 4:** Ionic Conductivity at Room Temperature (*T* = 25 °C) of the TPS_DIL Films at Different RH Conditions (RH 55%, 69%, and 84%)

sample	σ (S/cm)^a^ RH 55%	σ (S/cm) RH 69%	σ (S/cm) RH 84%
TPS_D1	(5.0 ± 0.2) × 10^–7^	(4.5 ± 0.1) × 10^–6^	(2.7 ± 0.1) × 10^–5^
TPS_D2	(4.0 ± 0.1) × 10^–6^	(3.7 ± 0.1) × 10^–5^	(8.4 ± 0.1) × 10^–4^
TPS_D3	(1.4 ± 0.1) × 10^–6^	(2.5 ± 0.1) × 10^–5^	(2.4 ± 0.2) × 10^–4^
TPS_D4	(3.7 ± 0.1) × 10^–6^	(2.1 ± 0.1) × 10^–5^	(3.3 ± 0.5) × 10^–5^

a Values obtained from the graph in Figure 7.

All samples exhibited a clear increase in conductivity with increasing RH, indicating enhanced ionic mobility facilitated by water uptake.^37^ However, the conductivity of the TPS_D1 sample was generally the lowest among all films for all humidity conditions tested. As suggested by FTIR analysis and mechanical properties, this may be attributed to excessive interactions between the polymer matrix and the cation, as reported in the literature,^38^ which reduce cation mobility and, consequently, the film’s ionic conductivity.

The TPS_D4 sample, on the other hand, although initially following a trend comparable to that of the other films, showed a limited increase in conductivity from 69% to 84% RH, potentially due to moisture saturation.

In order to investigate the ionic conductivity transport mechanism and the potential phase transitions within the operational temperature range, EIS measurements were conducted at various temperatures and a RH of 55%. Generally, ion conductivity can follow two main relationships for the temperature dependence of polymer dynamics: in totally amorphous polymers, it mainly follows the Vogel–Tamman–Fulcher relationship, while in crystalline or semicrystalline regions, the temperature dependence of ion motion commonly follows an Arrhenius form.

Figure 7 shows the temperature dependence of ionic conductivity for the prepared films at RH 55% in the 25 °C–65 °C range, which is commonly adopted in the literature to identify the conduction mechanism in similar systems.^39–41^ The overall conductivity linearly increases with the temperature, indicating no phase transition in the explored temperature range. The increasing temperature aids the movement of polymer chain segments, generating more free volume in which ions can move.^42,43^ This phenomenon combines with the ion hopping mechanism,^44^ following the Arrhenius relationship (6) expressed as

6 $$\sigma =\sigma_{0}\exp\left(-\frac{E_{a}}{k_{B}T}\right)$$

where σ_0_ is the pre-exponential factor, *E*_a_ is the activation energy, and *k*_B_ is the Boltzmann constant.

**Figure 7:**
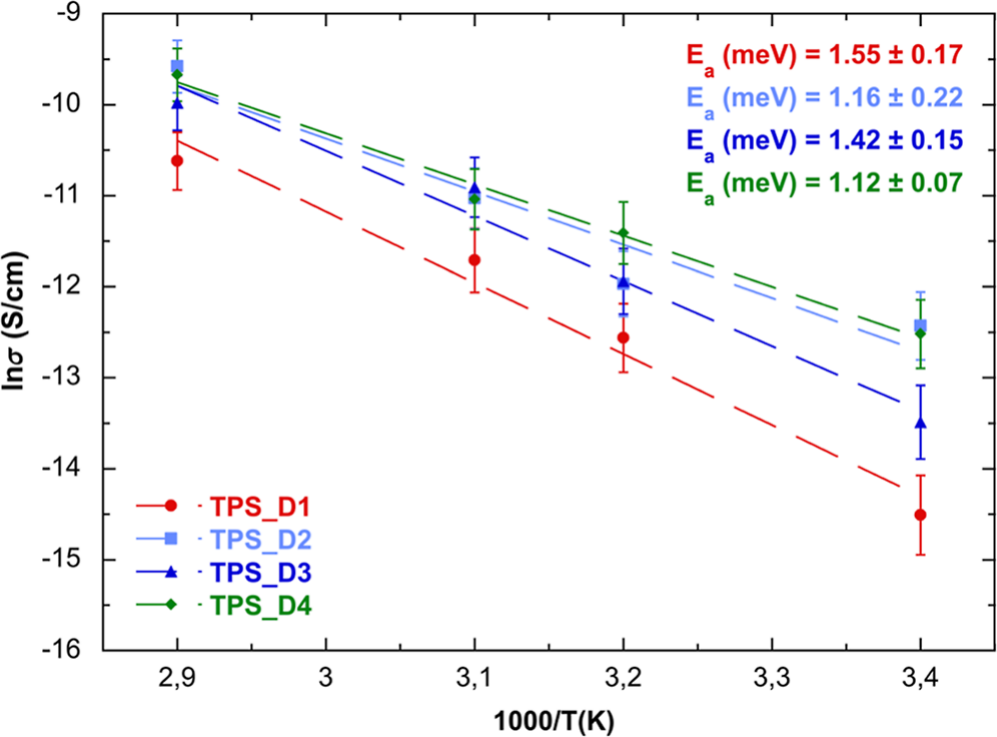
Variation of the conductivity as a function of temperature at RH 55%.

The *E*_a_ values, which are now associated with ion hopping, were obtained from the slope of the curves and are reported in Figure 7 (inset).

The Arrhenius plot confirms that the TPS_D1 sample exhibits slightly lower conductivity compared to the other films, which display comparable values.

Considering the complexity of the studied systems, as previously reported in the literature,^45–47^ there is no direct correlation indicating that high conductivity values correspond to low activation energy values.

### 3.9 Static Contact Angle Determination

In addition to good conductivity, hydrophobic stability is desirable for flexible electronics.^48^ The static water contact angle (θ) technique evaluates the surface wettability and interfacial interactions. This technique provides valuable insights into surface properties such as hydrophobicity and hydrophilicity by precisely measuring the angle formed between a water droplet and the film’s surface at equilibrium. Surfaces with water contact angles smaller than 90° are considered hydrophilic, while contact angles larger than or equal to 90° are classified as hydrophobic.^49^ Generally, water contact angle values for plasticized starch films lie between 20° and 70° and can be influenced by the roughness of the surface, the type of plasticizer used, film preparation methods, and storage conditions.^50–52^ In order to exclude the effect of roughness on the wettability results, the mean square heights (*S*_q_) and arithmetic mean heights (*S*_a_) of the surfaces were determined. The surface topography and roughness parameters of the different starch films are shown in Table 5. The TPS_DIL films exhibited comparable surface morphology, with *S*_a_ and *S*_q_ values falling within a similar range, allowing us to make a direct comparison between samples.^53–55^

**Table 5:**
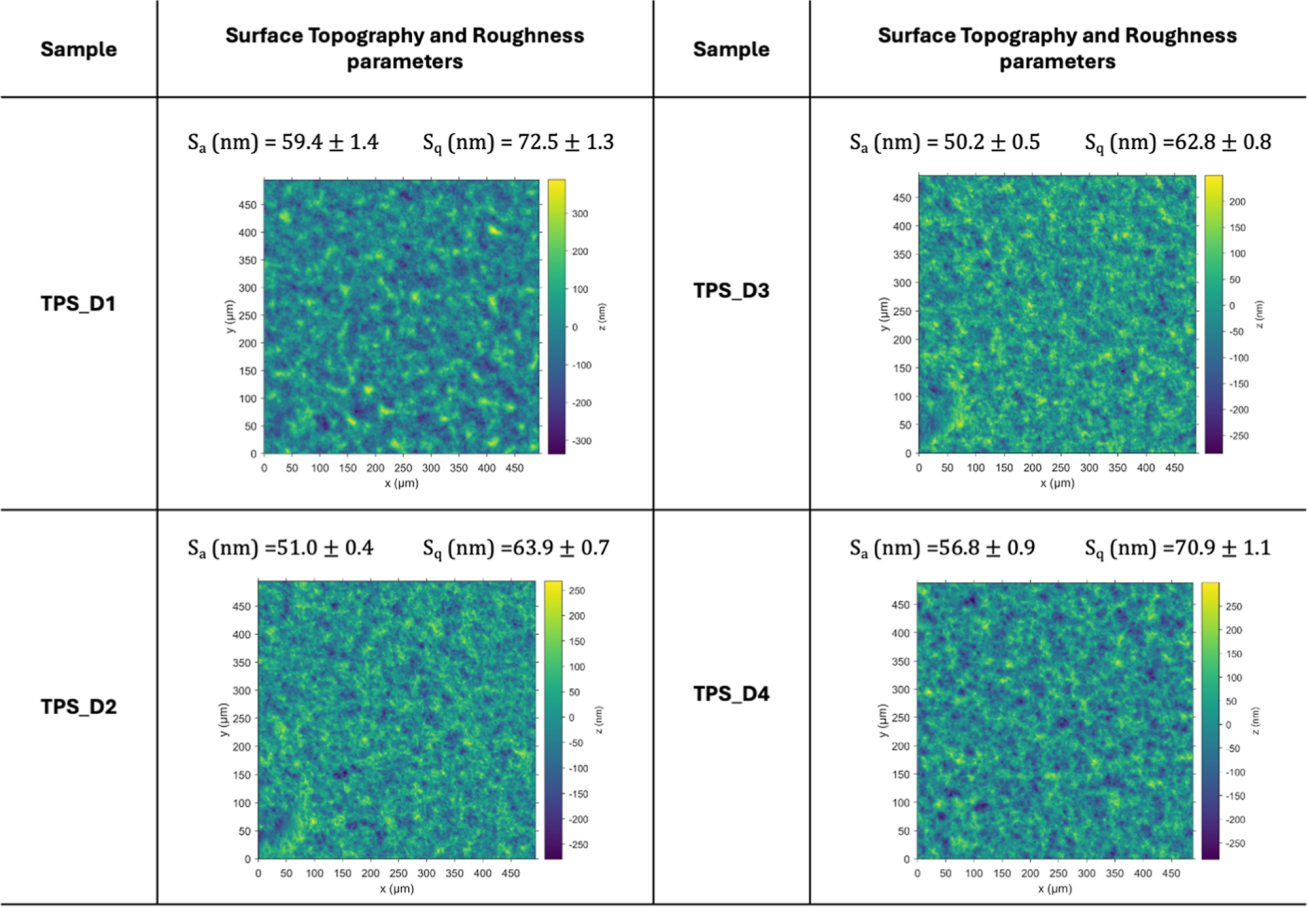
Surface Topography and Roughness Parameters of the TPS_DIL Films

The water contact angle values of the different starch films are shown in Figure 8. The plasticized films with C_1_H_2_(MIm)_2_Cl_2_ and C_6_H_4_(CH_2_MIm)_2_Cl_2_ show the highest contact angle values, 89° and 85°, respectively, pushing them to the limit of being defined as hydrophobic materials. The wettability also remains close to the hydrophobicity limit value when the plasticizer C_5_H_10_(MIm)_2_Cl_2_ is used (73°). Finally, when C_8_H_16_O_3_(MIm)_2_Cl_2_ is employed, the contact angle decreases to 52°. In general, as the linker chain length increases, a decrease in the contact angle is observed. This is likely due to the weakening of starch interchain interactions as the steric hindrance of the DIL grows. Consequently, the increased distance between starch chains reduces intermolecular hydrogen bonding, leading to a higher number of exposed −OH groups and, thus, enhanced hydrophilicity.^56^ However, the TPS_D3 sample deviates from this trend, displaying a higher contact angle of 85°, which is attributed to the more hydrophobic nature of its linker. Thus, the wettability of these materials is influenced not only by the linker chain length but also by its chemical nature.

**Figure 8:**
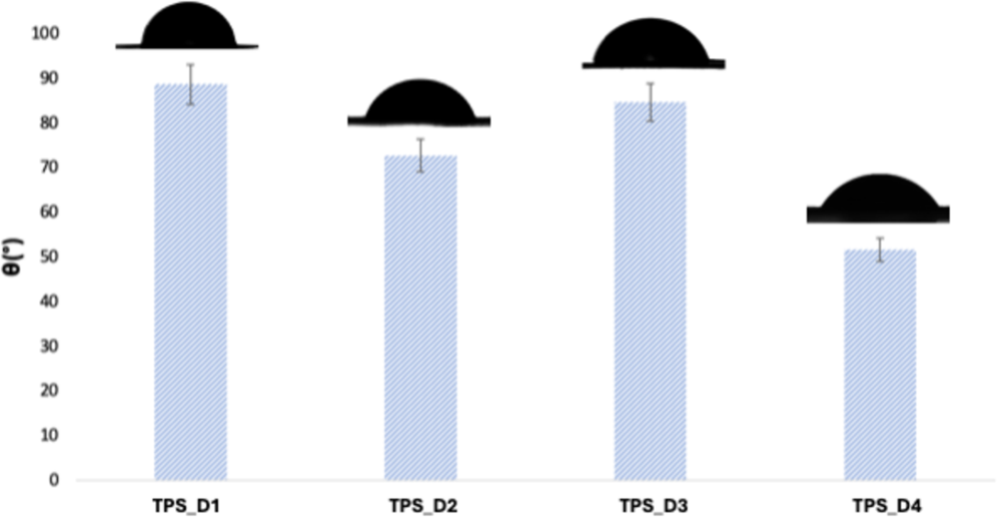
Water contact angle measurements and roughness parameters of TPS_DIL films.

### 3.10 Antimicrobial Activity

To investigate the antimicrobial activity of the starch films plasticized with different DILs, BGI experiments were performed on *S. epidermidis* (Gram-positive) and *P. aeruginosa* (Gram-negative), two of the most common bacteria associated with chronic infectious skin diseases and with infections on biomedical devices, responsible for up to 60% of all prosthetic infections.^16,57,58^ Although the exact bactericidal mechanism of DILs remains unknown, the main hypothesis involves the preferential adsorption of the cationic compound onto the negatively charged cell wall. This event is followed by the diffusion of the hydrophobic alkyl chains through the lipid bilayer, leading to the disruption of the cell membrane.^14,59^ As already reported in literature,^60^ the improved antibacterial efficiency of DILs compared to their monocationic counterparts could be attributed to their “bolaamphiphilic” structures.

Each film was able to inhibit the growth of the surrounding bacteria (Figure S11). As shown in Figure 9, the films were more effective against *S. epidermidis*, showing bacterial inhibition of up to 51%. On the contrary, they exerted a lower BGI on *P. aeruginosa*. These results may be related to the additional lipopolysaccharide layer present on the Gram-negative membrane, which hinders its permeation.^61^ It is worth noting that a low amount of DIL with respect to its optimal inhibitory concentrations (Table S2) is present in the films, as it was primarily introduced for plasticizing purposes. Generally, the higher the hydrophobicity of the cationic alkyl chain, the greater the antimicrobial activity.^16^ In agreement with this, TPS_D1 and TPS_D3 showed higher antibacterial activity against *P. aeruginosa*. Furthermore, the behavior of TPS_D3 is consistent with a study showing that the presence of an aromatic ring in the molecular structure of the DIL allows for greater diffusion through the lipid membrane of bacteria.^14^

**Figure 9:**
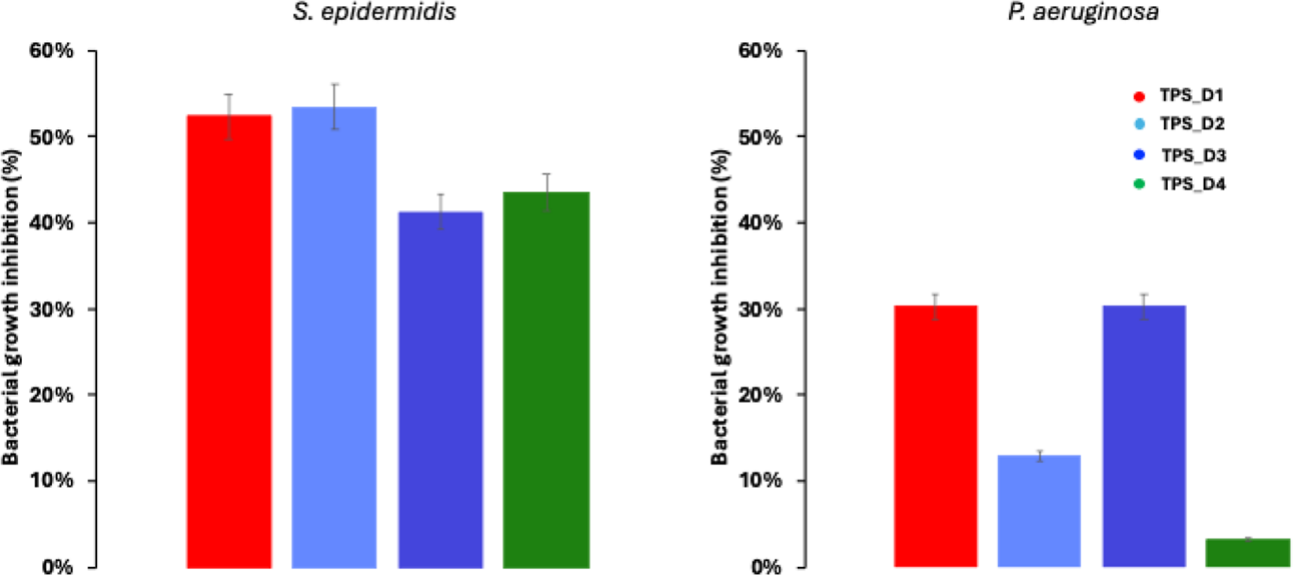
Bacterial growth inhibition histograms of TPS_DIL films tested on *S. epidermidis* and *P. aeruginosa*

### 3.11 Stimulus-Responsiveness of the Films

In the context of flexible electronics, the devices attracting the most interest are wearable sensors, which can detect and transmit a wide range of physical stimuli, such as strain, pressure, temperature, humidity, and more.^62,63^ Prepared starch-based films plasticized with DILs exhibit potential application prospects in strain sensor devices due to their mechanical properties, conductivity, and antibacterial characteristics. Regarding the last aspect, it is worth noting that wearable devices contact the environment of body fluids and sweat, making it easy for bacteria to proliferate, causing some skin diseases. Thus, using materials with intrinsic antibacterial properties is a significant advantage. The electromechanical properties were therefore tested to demonstrate the potential utility of these films as foundational materials for wearable sensor technology.^64^ Specimen 5 cm long and 0.5 cm wide were attached to an index finger and subjected to six repeated finger bending cycles. Figure 10 presents the percentage of relative resistance change (Δ*R*/*R*_0_) during the bending motion of the finger. The bending and relaxing of the finger are clearly detectable through the observed increase and decrease in relative resistance change for all of the films. In addition, it is noted that the resistance decreased when the finger began to bend instead of increasing, as observed for other common conducting materials, such as silver, copper, and carbon. This opposite behavior of conductivity to strain aligns with previous observation in other conducting materials containing ILs.^65^ This consistency in performance over repeated use underscores the reliability of these materials in long-term applications.

**Figure 10:**
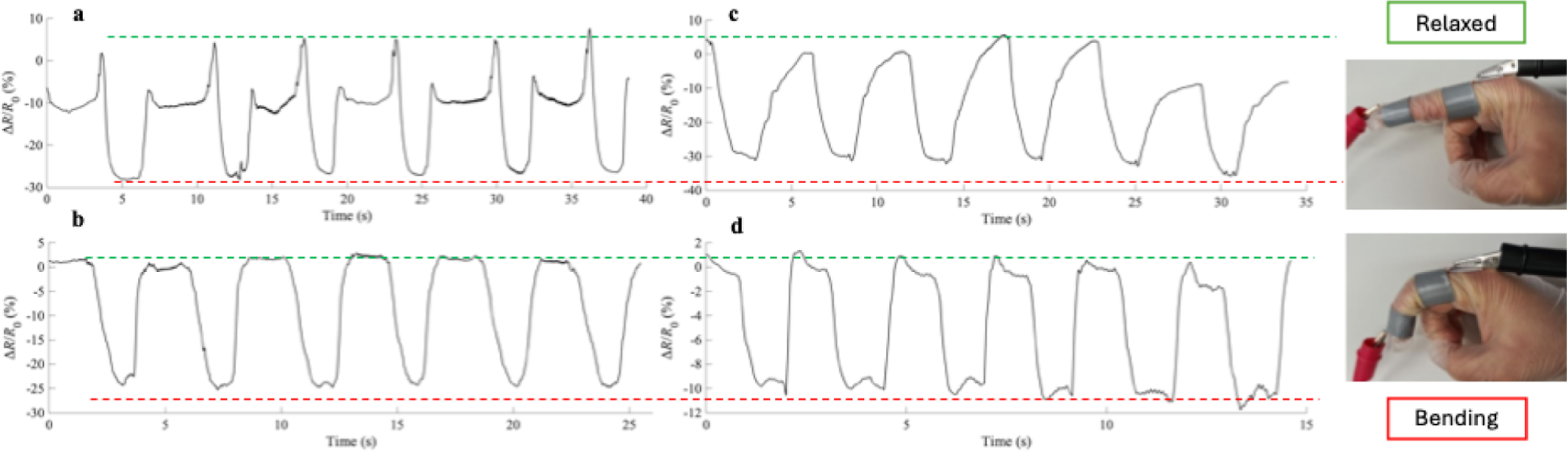
Response of (a) TPS_D1, (b) TPS_D2, (c) TPS_D3, and (d) TPS_D4 strain sensor in monitoring finger bending under six bending/relaxation cycles (insets show photographs of index finger motion).

For all the films, it can be observed that there are no significant differences in the relative resistance change between the first and the last bending cycle, indicating their good durability.^66^ Only the TPS_D1 sample showed a lower sensitivity to movement, failing to register the return to the relaxed finger accurately. TPS_D2 and TPS_D4 showed the most stable and identical responses, representative of good reliability as detectors.^67^

## 4 Conclusion

In this study, we successfully prepared starch films using a solution casting method with four different 1-ethyl-3-methyl imidazolium-based dicationic liquids (C_1_H_2_(MIm)_2_Cl_2_, C_5_H_10_(MIm)_2_Cl_2_, C_6_H_4_(CH_2_MIm)_2_Cl_2_, and C_8_H_16_O_3_(MIm)_2_Cl_2_) as plasticizers. We explored the development of starch-based films incorporating DILs as plasticizers, aiming to combine conductive and antibacterial properties for potential applications in flexible electronics. Our investigation focused on four distinct DILs based on 1-ethyl-3-methyl imidazolium, containing different alkyl chain lengths and chemical structures, to understand their impact on film properties. The findings from the FTIR analysis confirmed that the ILs did not react with starch, but their presence influenced the hydrogen bonding within the starch matrix, as evidenced by shifts in the O–H stretching bands. Thermal analysis indicated that the thermal stability of the films was consistent across samples, primarily determined by the anion rather than the cation of the DIL, while the glass transition temperature of the films was directly related to the cationic structure. Mechanical testing showed a direct correlation between the cationic structure and the mechanical behavior of the films as well. Increased steric hindrance and decreased structural mobility of the cations resulted in reduced TS and Young’s modulus, enhancing film flexibility. Electrochemical studies further elucidated the conductive nature of the starch films, with the ion diffusivity influenced by the alkyl chain lengths of the DILs. Despite the differences in conductivity being negligible, all films exhibited suitable electrochemical stability windows for potential use in flexible electronic devices, maintaining their performance even under varying humidity conditions. The results obtained demonstrated that the films’ properties, analyzed using the previously mentioned techniques, depend primarily on the linker chain length rather than its chemical nature. Conversely, static contact angle measurements indicated that the chemical structure of the linker mainly affects the wettability of the film. It was found that C_1_H_2_(MIm)_2_Cl_2_ and C_6_H_4_(CH_2_MIm)_2_Cl_2_ imparted hydrophobicity to the films, a desirable trait for applications in wearable electronics. Antibacterial assays demonstrated varying degrees of microbial inhibition, underscoring the potential of DILs to enhance the antimicrobial properties of starch films, particularly against Gram-positive bacteria. The film’s performance in strain sensor tests highlighted their potential utility in wearable technology, with TPS_D2 and TPS_D4 exhibiting the most stable and reliable responses during repeated bending cycles, making them particularly promising, although further research is required.

Overall, this work showcases the dual functionality of starch films with conductive and antibacterial properties, enabled by DIL plasticizers. These films present a promising, sustainable alternative for use in flexible electronics, particularly in applications requiring biodegradable and eco-friendly materials. Future research could explore optimizing these properties further and expanding the range of potential applications for these innovative starch-based films.

## Supplementary Material



## Notes

^1^H NMR and ^13^C NMR spectra of 1,5-bis­(1-methyl-1*H*-imidazole-3-ium) pentane dichloride, 3,3′-bis­(1-methyl-1*H*-imidazole-3-ium), 1,3-phenylenedimethylene dichloride, and 1,11-bis­(1-methyl-1H-imidazole-3-ium) (3,6,9-trioxaundecane) dichloride; set up of the starch film specimens attached to the forefinger; images of the appearance of the films; weight loss percentage after 7 days of immersion in artificial sweat solution; FT-IR spectra of the starch powder and dicationic imidazolium-based ILs; stress–strain curves of the prepared films; image showing test tubes with produced films in contact with the bacterial suspension after 24 h of incubation and controls; and MIC values of DILs on *S. epidermidis* and *P. aeruginosa* (PDF)
