# Supplementary material for: Dicationic Ionic Liquids as Antibacterial and Conductive Plasticizers: Effect of Cationic Structures on Starch Film Properties for Flexible Electronics
Source: ACS Appl Bio Mater. 2025 Aug 30;8(9):8297–309. doi: 10.1021/acsabm.5c01229 (PMC12442073; doi:10.1021/acsabm.5c01229)
Supplement: Supplementary file 1 [file mt5c01229_si_001.pdf]

Supporting Information

Dicationic Ionic Liquids as Antibacterial and Conductive Plasticizers: Effect of Cationic Structures on Starch Film Properties for Flexible Electronics

Susanna Romano[1], Benedetta Brugnoli[2], Serena De Santis[1], Daniele Rocco[1], Chiara Frezza[1], Giovanni Sotgiu[1], Giorgia Fiori[1], Gabriele Bocchetta[1], Salvatore Andrea Sciuto[1], Andrea Scorza[1], Irene Bavasso[3], Alessandro Stuart Savoia[1], Monica Orsini[1,*]

[1]Department of Industrial, Electronic and Mechanical Engineering, Roma Tre University, Via Vito Volterra 62, 00146 Rome, Italy

[2]Department of Chemistry, University of Rome “La Sapienza”, Piazzale Aldo Moro 5, 00185, Rome, Italy

[3]Department of Chemical Engineering Materials Environment, University of Rome “La Sapienza”, 00184 Roma, Italy

Corresponding author e-mail: monica.orsini@uniroma3.it

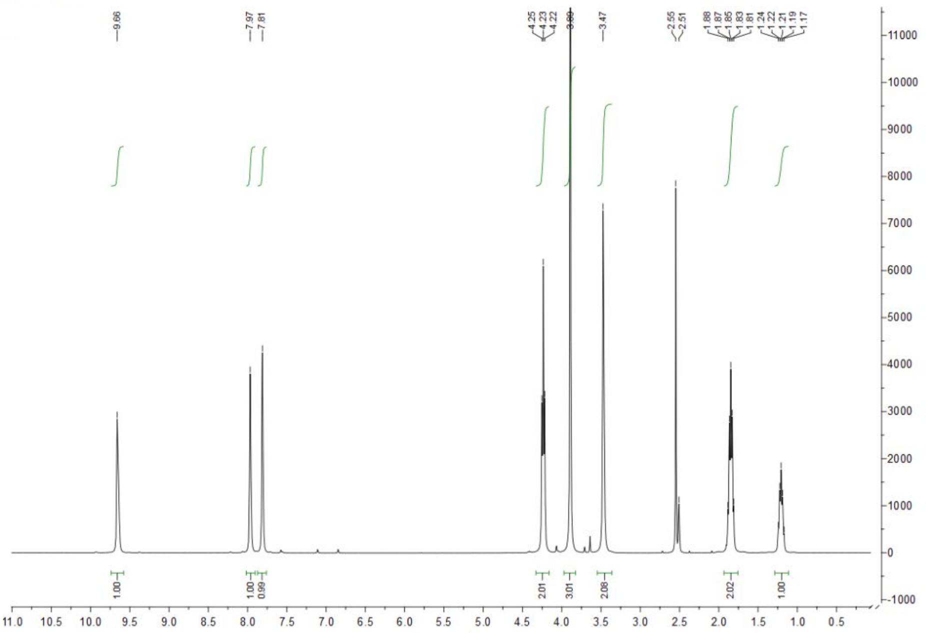

Figure S1. ¹H-NMR Spectra of 1,5-bis(1-methyl-1H-imidazol-3-ium) pentane dichloride

¹H NMR (400 MHz, DMSO)δ_{ppm}: 9.66 (s, 2H), 7.97 (s, 2H), 7.81 (s,2H), 4.25-4.22 (m, 4H), 3.89 (s, 6H), 1.88-1.81 (m, 4H), 1.24-1.17 (m, 2H) ppm

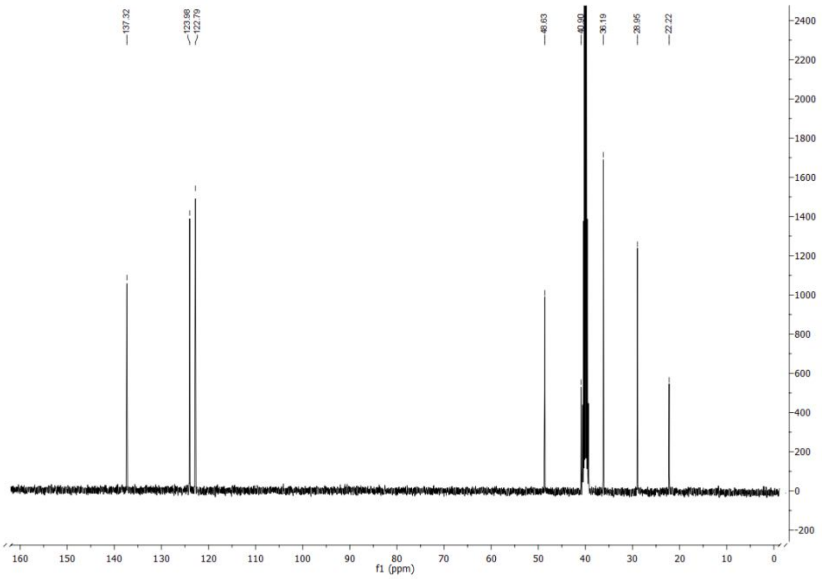

Figure S2. ^{13}C-NMR Spectra of 1,5-bis(1-methyl-1H-imidazol-3-ium) pentane dichloride

^{13}C NMR (400 MHz, DMSO)δ_{ppm}: 137.32, 123.98, 122.79, 48.83, 36.19, 28.95, 22.22 ppm

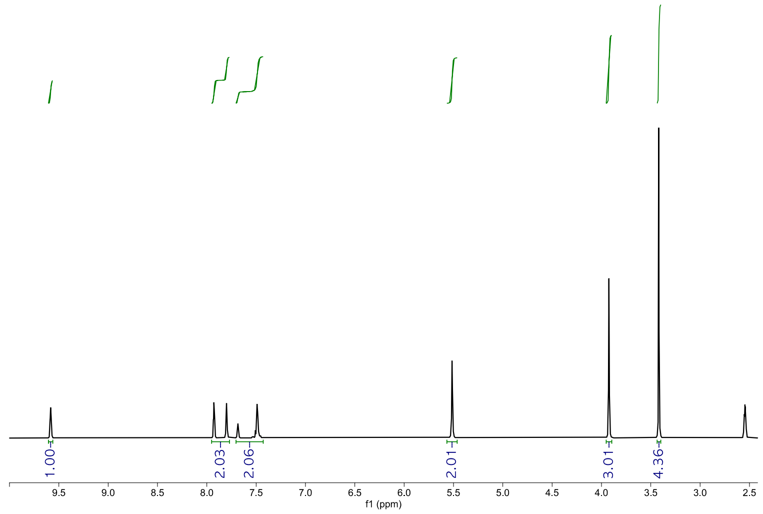

Figure S3. ^{1}H-NMR Spectra of 3,3'-bis(1-methyl-1H-imidazol-3-ium) 1,3-phenylenedimethylene dichloride

^{1}H NMR (400 MHz, DMSO)δ_{ppm}: 9.67 (s, 2H), 8.02 (s, 2H), 7.96 (s, 2H), 7.61–7.65 (m, 2H), 7.49–7.53 (m, 2H), 5.92 (s, 4H), 4.05 (s, 6H) ppm

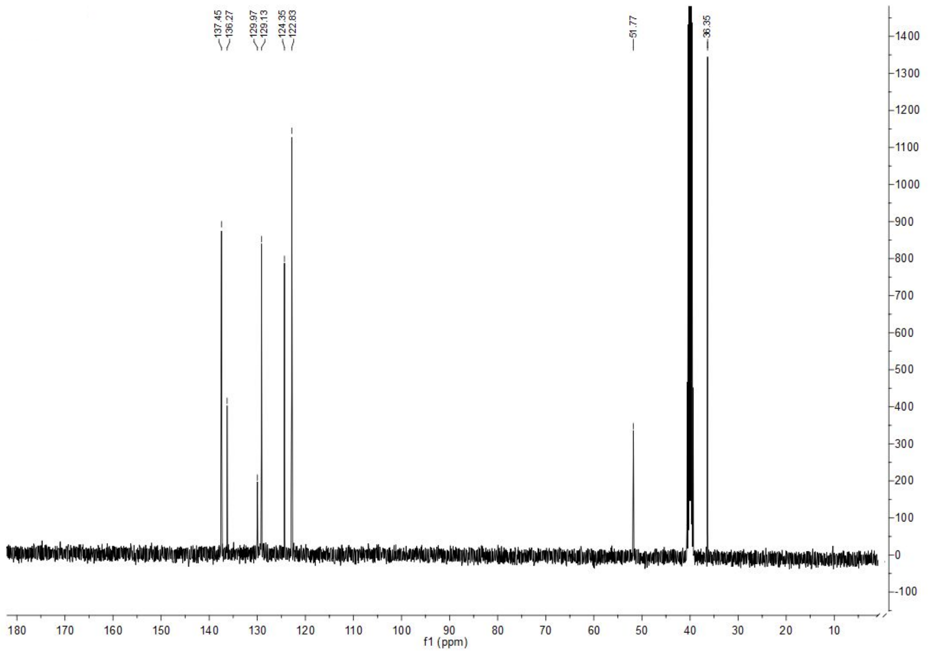

Figure S4. ^{13}C-NMR Spectra of 3,3'-bis(1-methyl-1H-imidazol-3-ium) 1,3-phenylenedimethylene dichloride

^{13}C NMR (400 MHz, DMSO)δ_{ppm}: 137.45, 136.27, 129.97, 129.13, 124.35, 122.83, 51.77, 36.35 ppm

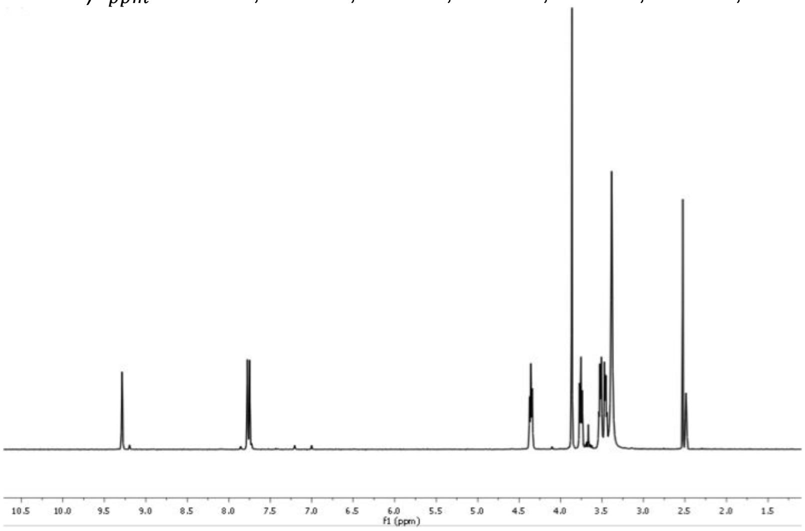

Figure S5. ^{1}H-NMR Spectra of 1,11-bis(1-methyl-1H-imidazol-3-ium) (3,6,9-trioxaundecane) dichloride

^{1}H NMR (400 MHz, DMSO)δ_{ppm}: 9.3 (s, 2H), 7.78 (s, 2H), 7.75 (s, 2H), 4.37 (t, 4H), 3.88 (s, 6H), 3.77 (t, 4H), 3.55–3.52 (m, 4H), 3.48–3.46 (m, 4H).

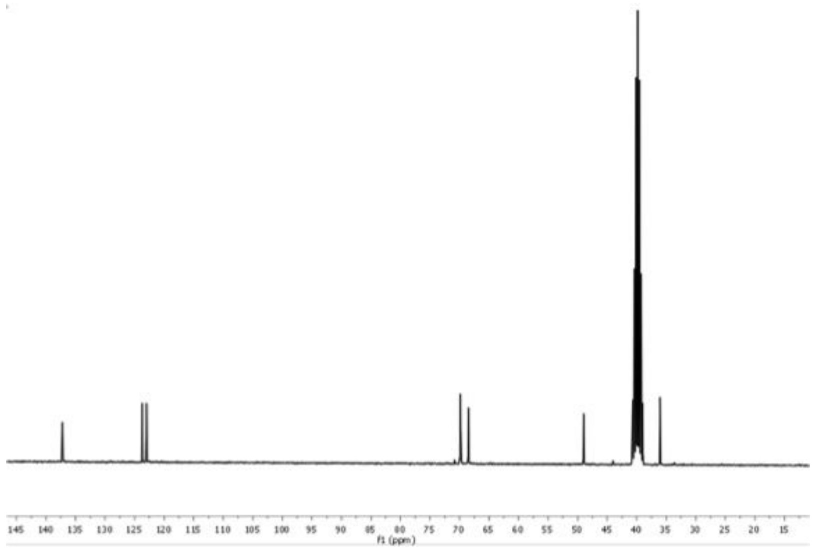

Figure S6. ^{13}C-NMR Spectra of 1,11-bis(1-methyl-1H-imidazol-3-ium) (3,6,9-trioxaundecane) dichloride

^{13}C NMR (400 MHz, DMSO)δ_{ppm}: 136.70, 123.12, 122.43, 69.35, 67.99, 48.51, 35.56

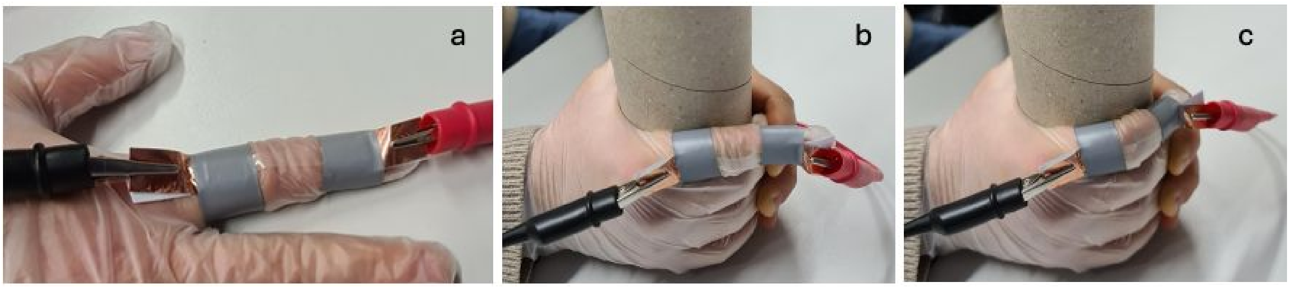

Figure S7. (a) Set up of the starch film specimens attached to the forefinger, (b) relaxation position, and (c) maximum bending position

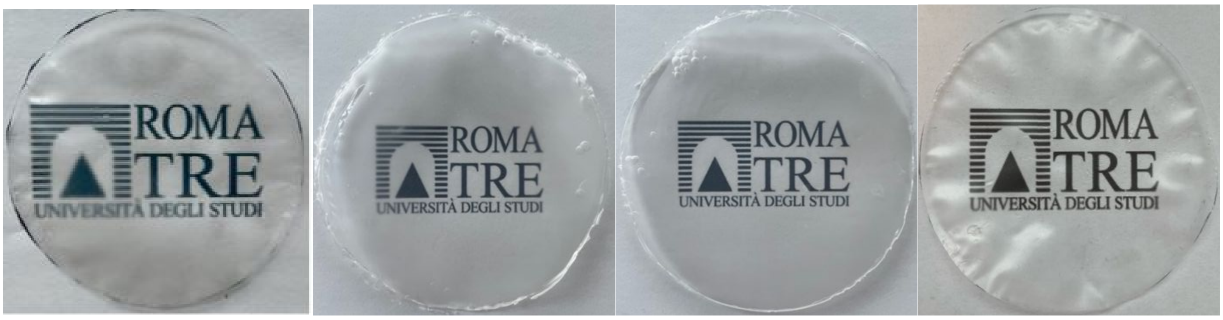

Figure S8. Digital images of the TPS_DIL films, from the left: TPS_D1, TPS_D2, TPS_D3, TPS_D4

Table S1. Weight loss percentage after 7 days of immersion in artificial sweat solution

**Acronym**	**Weight loss %**
TPS_D1	20 ± 3
TPS_D2	23 ± 1
TPS_D3	18 ± 1
TPS_D4	26 ± 1

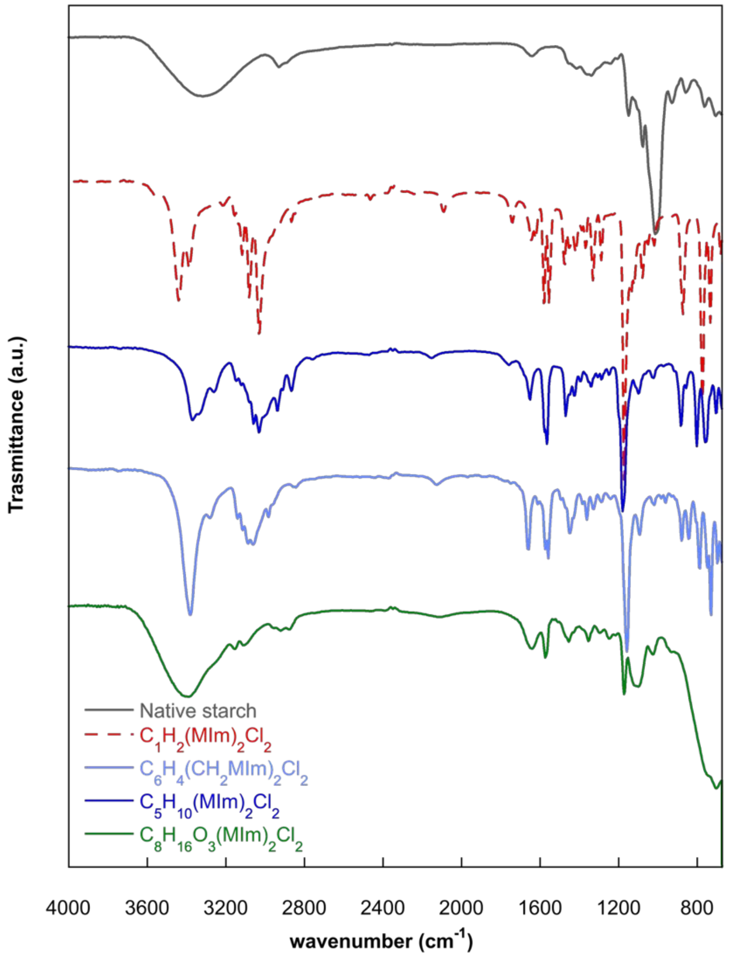

Figure S9. FTIR spectra of the starch powder and DILs

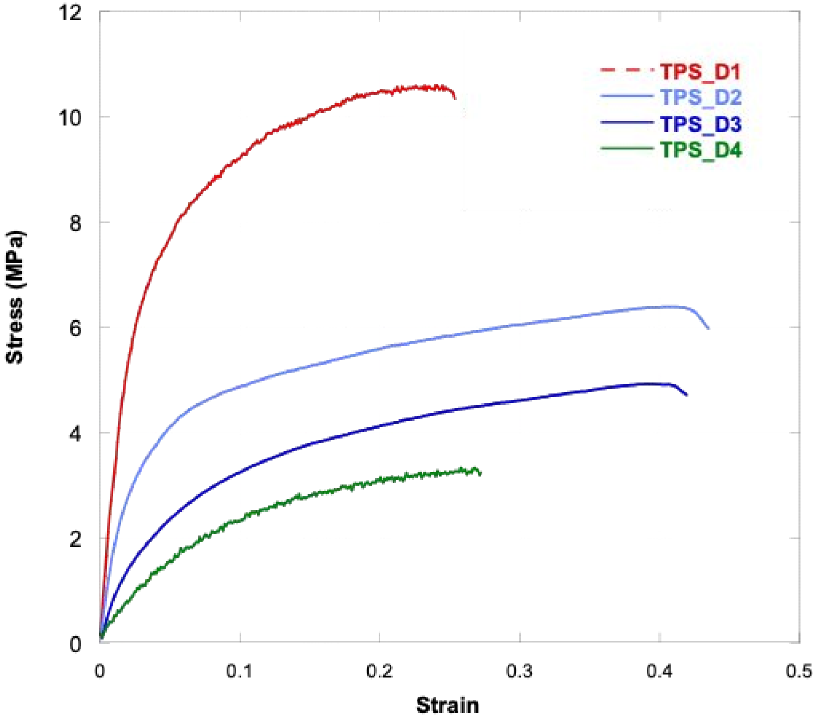

Figure S10. Stress-strain curves of the TPS_DIL samples

Table S2. MIC values of DILs on S. epidermidis and P. aeruginosa

	**MIC (mM)**	
**Sample**	***S. epidermidis***	***P. aeruginosa***
$C_1H_2(MIm)_2Cl_2$	201	25
$C_5H_{10}(MIm)_2Cl_2$	91	11
$C_6H_4(CH_2MIm)_2Cl_2$	40	10
$C_8H_{16}O_3(MIm)_2Cl_2$	144	36

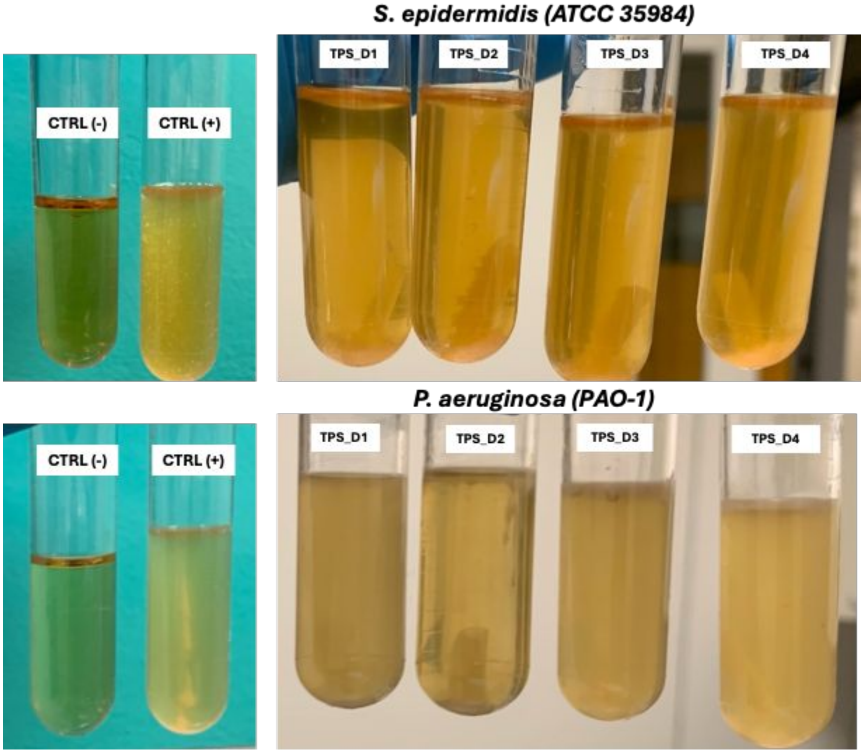

Figure S11. BGI test: image showing test tubes with negative and positive controls (left) and TPS_DIL (right) in contact with the bacterial suspension after 24h of incubation
